# Differential sensitivity of MCPH1- and BRCA2-deficient cancer cells to PARP-1 inhibition

**DOI:** 10.1371/journal.pone.0345514

**Published:** 2026-04-03

**Authors:** Isobel G. Chapman, Xueqin Wu, Stephany Veuger, Paul A. Jowsey

**Affiliations:** School of Geography and Natural Sciences, Northumbria University, Newcastle upon Tyne, United Kingdom; Zhejiang Cancer Hospital, CHINA

## Abstract

Microcephalin-1 (MCPH1) is a tumour suppressor protein that regulates homologous recombination repair (HRR) and is down-regulated in several tumour types. Given that HRR-defective cancer cells can be killed via synthetic lethal approaches, MCPH1 thus represents an attractive target in cancer therapy. Functionally, cells lacking MCPH1 have reported defects in the recruitment and retention of BRCA2 and RAD51 to DNA double strand breaks (DSBs) during HRR, though the magnitude of this defect in human cells is not entirely clear. Multiple studies have demonstrated that HRR-defective cells, particularly those lacking BRCA1 and BRCA2, can be specifically killed by inhibitors of the base excision repair enzyme, poly(ADP-ribose) polymerase-1 (PARP-1). Mechanistically, PARP-1 inhibition can cause (i) elevated DNA single strand breaks (SSBs) and (ii) ‘PARP-1 trapping’ on damaged DNA, both of which can lead to the formation of DSBs during DNA replication, which would normally be repaired by HRR. Given the functional link between MCPH1 and BRCA2, this study aimed to compare HRR-deficiency in cells lacking either protein and correlate this with PARP-1 inhibitor sensitivity. Our data shows that MCPH1-deficient cells are defective in HRR but still retain ~50% activity and this results in little to no sensitivity to two clinically-relevant PARP-1 inhibitors. In contrast, BRCA2-deficient cells showed a far greater defect in HRR and consistent sensitivity to both PARP-1 inhibitors, which was not enhanced by co-depletion of MCPH1. These data suggest that the magnitude of HRR defect in cancer cells influences PARP-1 inhibitor sensitivity and BRCA2 retains significant functionality in the absence of MCPH1.

## Introduction

Microcephalin-1 (MCPH1) is a tumour suppressor protein, with roles in cell cycle regulation, chromatin remodelling and DNA repair [1,2]. Structurally, MCPH1 is a 835-amino acid protein, with a single BRCT domain at its N-terminal and a pair of BRCT domains at its C-terminal. The N-terminal and central region of the protein is generally involved in chromosome condensation via regulation of the condensin II complex, whereas the C-terminal BRCT domains interact with a range of DNA damage response (DDR) proteins, including histone H2AX, BRCA2 and RAD51. The interaction with H2AX is important for the initial recruitment of MCPH1 to sites of DNA damage and is mediated by the binding of MCPH1 BRCT domains to phospho-serine 139 of H2AX [3]. Once at the site of damage, MCPH1 has been implicated in the recruitment of other DDR factors, including MDC1, 53 BP1, TOPBP1, BRCA2, RAD51 and RAD17, thus contributing to various DNA damage response pathways [4–6].

The BRCA2-RAD51 complex is a key component for the repair of DNA double strand breaks (DSBs) via homologous recombination repair (HRR) [7,8]. When DSBs are generated, for example via ionising radiation or drugs with radiomimetic action, the DSB is initially recognized via the MRE11-RAD50-NBS1 (MRN) complex, which binds and activates the ATM protein kinase. A key target of ATM is histone H2AX, with phosphorylation on Ser139 forming a binding platform for other proteins, including BRCA1, that promotes 5’-end-resection at the DSB. End resection drives DSB repair pathway choice towards HRR, rather than the less accurate non-homologous end joining (NHEJ), and the resultant single-stranded stretches of DNA are immediately coated by Replication Protein A (RPA), which is subsequently replaced by RAD51 in a BRCA2-dependent manner. The RAD51-coated ‘nucleofilaments’ are then able to invade a sister chromatid and align with homologous DNA sequences, initiating error-free repair of the damaged chromosome.

Cells lacking MCPH1 have decreased HRR and there are multiple levels at which MCPH1 has been implicated in this pathway, either directly or indirectly. For example, (i) regulation of BRCA1 gene expression and thus protein levels [9,10], (ii) as part of a complex with condensin II that regulates chromosome compaction [11] and (iii) as a key factor in the recruitment and retention of the BRCA2-RAD51 complex to DSBs. Most evidence relates to the latter mechanism, though there is a lack of quantitative analysis of BRCA2/RAD51 function in human cells lacking MCPH1 and no direct comparison of HRR efficiency in MCPH1- versus BRCA2-deficient cells. Wu *et al* (2009) demonstrated that BRCA2 and RAD51 co-immunoprecipitated with MCPH1 and depletion of MCPH1 using siRNA caused a defect in BRCA2 and RAD51 foci formation after ionising radiation (IR) [5]. An MCPH1 mouse knockout (KO) study demonstrated a quantitative defect in RAD51 foci formation during meiotic recombination in isolated MCPH1 KO spermatocytes [12]. The same study also demonstrated a defect in the formation of BRCA2 and RAD51 foci after IR in MCPH1 KO mouse embryonic fibroblasts. Finally, a recent study used an elegant cell-free system to demonstrate that MCPH1 was required for the retention of RAD51 on single stranded nucleofilaments, though this system lacks the regulatory complexities of cell-based chromatin structure and the HRR pathway [13].

There is an abundance of evidence that cells lacking BRCA2 (and BRCA1) are killed by inhibitors of poly(ADP-ribose) polymerase-1 (PARP-1) via a synthetic lethality mechanism, stemming from two seminal studies in 2005 [14,15]. This interaction is now exploited in the clinic, with PARP-1 inhibitors approved for the treatment of breast, ovarian, prostate and pancreatic tumours harbouring mutations in BRCA1 or BRCA2 [16–19]. Importantly, MCPH1 has also been shown to be down-regulated in breast, ovarian and prostate tumours. For example, MCPH1 analysis by immunohistochemistry revealed down-regulation in 29% of breast cancers and 33% of ovarian cancers [20,21]. In addition, comparative genome analysis showed reduced MCPH1 gene copy number in 40% of ovarian tumour samples and 72% of breast cancer cell lines [4]. Given the role of MCPH1 in HRR, it is possible that these MCPH1-deficient tumours will be sensitive to PARP-1 inhibitors.

Mechanistically, PARP-1 inhibition has been proposed to cause (i) increased DNA SSBs via blocked base excision repair (BER) and/or (ii) ‘PARP-1 trapping’ on damaged DNA, due to lack of PARP-1 autoribosylation [22,23]. Both effects lead to the formation of DSBs during DNA replication and thus HRR-defective cells would be expected to be more sensitive to PARP-1 inhibitor monotherapy. Whilst the sensitivity of BRCA2-deficient (and BRCA1-deficient) cells is widely recognised, the evidence regarding other components of HRR is less clear, particularly in the clinic. For example, several recent clinical trials investigated the impact of PARP-1 inhibitors in HRR-deficient cancers and demonstrated a clinical benefit for patients with BRCA1 and BRCA2 mutations but not for other components of HRR, including ATM, BARD1, PALB2 and RAD51 (among others) [16,24]. It is likely that the degree of HRR-deficiency varies, depending upon the component of HRR that is mutated/absent and this potentially impacts PARP-1 inhibitor sensitivity. We therefore decided to explore this possibility by focusing on the functional relationship between MCPH1 and BRCA2 by quantifying DSB repair and HRR in MCPH1- and BRCA2-deficient cells and correlating this with PARP-1 inhibitor sensitivity. Interestingly, our data showed a partial HRR defect in MCPH1-deficient cells and little/no evidence of PARP-1 inhibitor sensitivity. In contrast, BRCA2-deficient cells had a more marked HRR defect and consistent sensitivity to PARP-1 inhibitors. These findings add to the clinical debate over the utility of PARP-1 inhibitors in treating HRR-deficient cancers with non-BRCA mutations and also suggest that BRCA2 retains significant HRR functionality in cells lacking MCPH1.

## Materials and methods

### Cell culture and drugs

HeLa (Cervical carcinoma),U2OS (osteosarcoma) and HEK293 cell lines were obtained from UK Health Security Agency Culture Collections and cultured in DMEM containing 10% fetal bovine serum and 2mM L-glutamine (Gibco). Cells were maintained as exponentially growing cultures at 37°C in a humidified atmosphere with 5% CO_2_. HEK293 cells were used to generate an MCPH1-knockout cell line using CRISPR-CAS9. An annealed MPCH1-targeting primer was inserted into plasmid pEsgRNA (MRC PPU Reagents and Services, University of Dundee) using insertion mutagenesis [25]. The primer sequence was 5’-GGAAAGGACGAAACACCGGGCCGCCATGACAGACGGCGTTTTAGAGCTAGAAAT-3’ and resultant plasmid designated pEsgMCPH1. HEK293 cells were transfected with pEsgMCPH1 and GFP-CAS9 (kindly provided by Antony Antoniou, Northumbria University) using polyethyenimine (Merck). Control cells were transfected with pEsgRNA and GFP-CAS9. Forty eight hours after transfection, cells were seeded as single cells in 96-well plates and colonies allowed to form, before analysis of MCPH1 knockout using Western blotting. Cells lacking MCPH1 expression were designated sgMCPH1 and control cells (expressing normal levels of MCPH1) designated sgCON. PARP-1 inhibitors, AZD-2461 (Tocris) and Talazoparib (MedChemExpress) were prepared at stock concentrations of 10mM and 50mM, respectively in DMSO. Final concentrations of PARP-1 inhibitors are indicated in the relevant experimental data, with DMSO concentrations maintained at 0.1% (v/v) in all samples, unless otherwise stated. Etoposide (Merck) was prepared as a 50mM stock in DMSO and used at the indicated final concentrations (with DMSO concentrations maintained at 0.1% v/v).

### SiRNA transfections

To target MCPH1 using small-interfering RNA (SiRNA) the following sequence was used; AAAGGAAGTTGGAAGGATCCA (siMCPH1, synthesised by Qiagen). BRCA2 was targeted using SMARTpool siGENOME Human BRCA2 (675) siRNA [(Horizon Discovery Bioscience, M-003462-01-0005)(siBRCA2)]. All siRNA experiments included a non-targeting control – AllStars Negative control siRNA (Qiagen, siCON). For transfection, 2 x 10^5^ cells were seeded into 6 cm plates. After 24 hours, cells were transfected using Lipofectamine RNAiMAX (Invitrogen). For each transfection, 150µl of OPTI-MEM medium (Gibco) was added to 2x 15 ml Falcon tubes. To one tube, 3.3µl of siRNA (from 10µM stock) was added, whilst to the other tube 7µl of Lipofectamine RNAiMAX (Invitrogen) was added. After mixing and incubation for 5-min, the tube contents were combined and incubated for a further 5-min before adding drop-wise to cells.

### Western blotting

Cells were lysed directly in 1X LDS sample buffer (Invitrogen) containing 0.2% (v/v) Universal Nuclease (Thermo Scientific) prior to heating at 70°C for 10 minutes. Protein concentrations were standardised to 2µg/µl using 1x LDS and 2.5% (v/v) 2-mercaptoethanol added to each sample. Protein electrophoresis was carried out using 3−8% Tris-Acetate or 4−12% bis tris NU-PAGE gels (Invitrogen) prior to transfer to nitrocellulose membrane using transfer stacks and an iBlot2 machine (both Invitrogen). Membranes were blocked with 5% dried skimmed milk/TBS-T (50mM Tris pH 7.6, 150mM NaCl and 0.2% v/v Tween-20) for a minimum of 1-hour prior to overnight incubation at 4°C with primary antibodies; MCPH1 (11962–1-AP, Proteintech), BRCA2 (29450–1-AP, Proteintech) and GAPDH (60004–1-Ig, Proteintech). Membranes were then washed with TBS-T and incubated with secondary antibody, Anti-rabbit HRP Conjugated (7074S, Cell signalling) for 2 hours at 4°C. After thorough washing of membranes in TBS-T, protein bands were visualised using ECL Select, Chemiluminescent Substrate (Cytiva Amersham) and images captured using a Syngene G:Box Chemi XX6 gel documentation system and quantified using associated GeneTool software (Syngene, version 4.03.020) or ImageJ.

### DAPI staining and microscopy for PCC phenotype

Cells were plated out on to coverslips and transfected with siCON or siMCPH1 for 48h (as previously described), prior to fixation with 3.7% formaldehyde (Fisher), washed with PBS and incubated with 0.2% Triton X-100 containing 0.2 µg/ml DAPI (Merck) for 10 minutes. Cells were washed with PBS and mounted on to microscope slides using Hydromount Mounting Media (National Diagnostics). Confocal fluorescence microscopy (Leica DMi8) was used to identify cells demonstrating PCC phenotype with tightly condensed chromatin within an intact nuclear membrane. At least 100 cells were analysed per slides and the percentage of PCC cells were scored.

### Immunofluorescence analysis of γ-H2AX

U2OS cells (1 x 10^4^) were seeded into 4-well culture slides (Thermo Scientific Nunc Lab-Tek) and transfected with either siCon, siMCPH1 or siBRCA2 for 48hours, as described above. Cells were treated with doses of Etoposide at 0, 1, 5 and 20µM for 5 hours. The medium was removed, and cells were washed with PBS, prior to fixation with 3.7% formaldehyde for 10 minutes. Cells were then washed three times with PBS and permeabilised with 0.2% (v/v) Triton X-100 (in PBS) for 20 minutes before three further PBS washes. Cells were then incubated with blocking buffer [PBS containing 5% goat serum (Merck) and 0.2% v/v Tween 20] for 1 hour, before incubation with primary antibody [phospho-H2AX Ser139 (83307–2-RR, Proteintech, 1:500) overnight at 4ºC. After three washes with PBS-T (PBS containing 0.2% v/v Tween 20), cells were incubated with secondary antibody (goat anti-rabbit Multi-rAb Coralite Plus 594, Proteintech, 1:500 in blocking buffer) for 2 hours, in the dark at 4ºC. Cells were then washed with PBS-T for 30 minutes prior to staining with 0.1µg/ml DAPI (in PBS) for ten minutes at room temperature. After three PBS washes, coverslips were then mounted on slides using Hydromount Mounting Media (National Diagnostics). Imaging and quantification (% of cells showing >5 H2AX foci) were carried out using fluorescence microscopy (Leica DMi8 Confocal Microscope), with 200 cells analysed per treatment.

### MTS assay

Cells were transfected with siCON, siMCPH1 or siBRCA2 for 48h (as described previously), before seeding into 96-well plates. Cells were treated with the indicated concentrations of PARP-1 inhibitor, with DMSO maintained at 0.1% (v/v) in all wells (each treatment was performed in triplicate). Cell viability was assessed after 96-hours using an MTS assay. MTS reagent (Promega) was added to each well (20% of well volume) and cells were incubated at 37ºC for up to 2 hours prior to reading absorbances of each well at 490nm. The average absorbance of blank wells (containing media only + MTS) was subtracted from sample absorbance readings prior to data analysis. Data from at least three independent repeats was combined. Cell viability was plotted as % relative to corresponding DMSO-treated cells, where these cells were designated as 100% relative cell viability.

### Colony formation assay

Cells were transfected with siCON, siMCPH1 or siBRCA2 for 48h (as described previously), before seeding into 6-well plates (100 cells per well) and treatment with the indicated concentrations of PARP-1 inhibitors. After 10–14 days of growth at 37°C, colonies were fixed with methanol, washed with PBS and stained with 1% (w/v) Methylene Blue (Fisher Scientific) for 10 minutes. Excess stain was removed by water washes and plates allowed to dry. Colonies were counted from three independent experiments and data was presented as percentage colony formation relative to corresponding DMSO-treated cells, where these cells were designated as 100% colony formation.

### HRR repair GFP-based reporter assays

DR-GFP-U2OS cells with doxycycline-inducible I-SceI were generated by Dr Owen Wells and kindly provided by Professor Tim Humphrey (Genome Damage and Stability Centre, University of Sussex) [26]. Cells were transfected with siCON, siMCPH1 or siBRCA2 for 48 hours. Cells were treated with 5 μg/ml Doxycycline (Merck) for a further 48 hours, then harvested via trypsinisation, washed with PBS and resuspended in 100μl PBS. Cells were fixed by adding 500μl of 3.7% formaldehyde for 15 minutes, before being centrifuged and resuspended in 1 ml PBS. Samples were analysed using a CytoFLEX SRT cell sorter (Beckman Coulter), 10^4^ events per sample were collected and % GFP positive expressing cells were quantified and analysed using Floreda.io software (https://floreada.io). Quantified data from 3 independent biological replicates were combined and mean % GFP positive expressing cells presented as bar charts relative to control cells.

### Statistical analysis

Quantitative data is expressed as the mean ± standard error from at least three independent experiments, unless otherwise stated. Data was analysed using two-way ANOVA or unpaired t-tests, as indicated. For all analyses, P < 0.05 was considered statistically significant.

## Results

### Generation of MCPH1- and BRCA2-deficient cells

**Fig 1:**
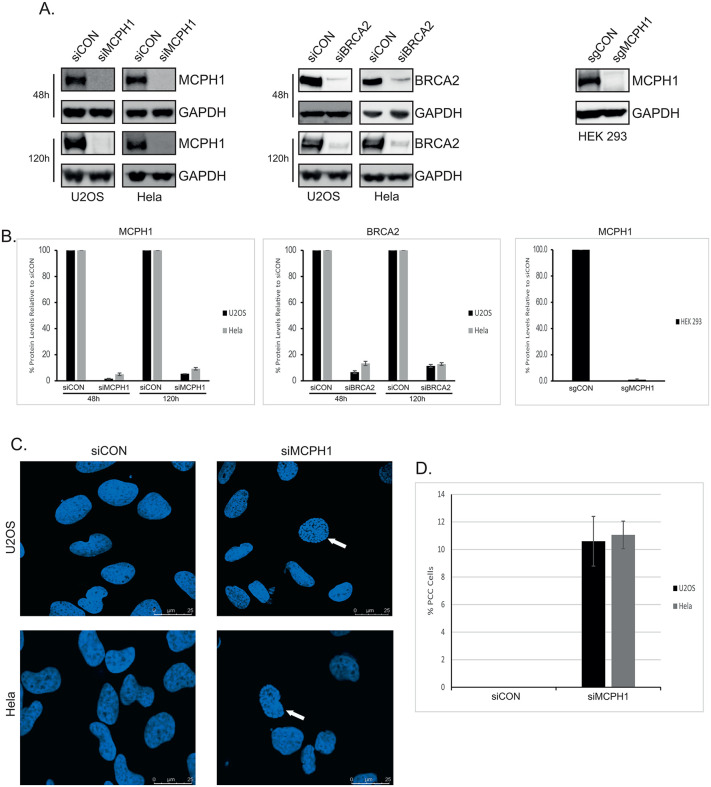
Generation of MCPH1- and BRCA2-Deficient Cells. **(A)**. U2OS or Hela cells were transfected with Control (siCON), MCPH1 (siMCPH1) or BRCA2 (siBRCA2) siRNA for 48h or 120h and protein expression analysed via Western blotting with the indicated antibodies. MCPH1 expression in sgCON (control) and sgMCPH1 (MCPH1 knockout) cells was also investigated by Western blotting. **(B)** Quantification of MCPH1 or BRCA2 protein bands normalised to GAPDH and expressed as % change relative to siCON or sgCON. Data is the mean and standard error of three (48h) or two (120h and sgCON/sgMCPH1) independent experiments. **(C)** U2OS or Hela cells were transfected with MCPH1 siRNA for 72h before analysis of nuclear morphology (premature chromosome condensation, PCC) via DAPI staining and fluorescence microscopy. **(D)** The % of cells displaying PCC, with at least 100 cells analysed.

To generate MCPH1- and BRCA2-deficient Hela and U2OS cells, siRNA was used and protein knockdown assessed using Western blotting. As showing in Fig 1A and 1B, 48h after siRNA transfection, MCPH1 levels were reduced by approximately 95% in both U2OS and Hela cells (siMCPH1). Efficient knockdown of BRCA2 was also observed (siBRCA2), with around a 90% decrease in U2OS and 95% decrease in Hela cells. Similar knockdown levels of both proteins were observed at 120h post-transfection, confirming long-term suppression. Western blotting also confirmed the lack of MCPH1 expression in CRISPR KO HEK293 cells (Fig 1A and 1B). Cells lacking MCPH1 have an unusual phenotype of premature chromosome condensation (PCC), demonstrated by an increase in the number of ‘prophase-like cells’ in asynchronous cultures. These cells have condensed chromosomes and an intact nuclear membrane (uniform nuclear perimeter, compared to irregular nuclear perimeter in normal prophase cells due to nuclear envelope breakdown). Therefore, to confirm that the knockdown of MCPH1 was functionally relevant, the percentage of PCC cells was quantified using fluorescence microscopy of DAPI-stained cultures. As shown in Fig 1C and 1D, knockdown of MCPH1 (siMCPH1) caused ~10% of PCC cells (Hela and U2OS), with no PCC cells observed in control cells (siCON), consistent with previously published studies.

### Cells lacking BRCA2 have a greater defect in HRR-mediated DSB repair compared to MCPH1-deficient cells

**Fig 2:**
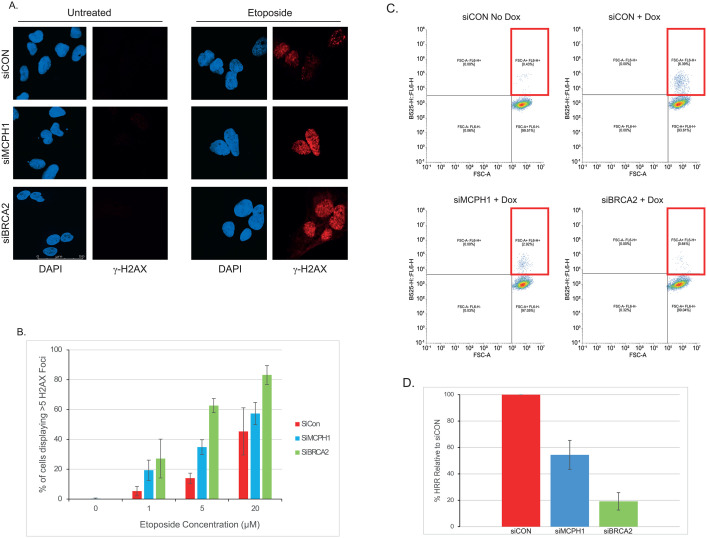
Continued.

Previous studies have suggested that MCPH1-deficient cells have a defect in HRR, mediated by aberrant recruitment/retention of BRCA2 and RAD51 to DNA DSBs. Given the putative functional link between MCPH1 and BRCA2, we directly compared both DSB repair and the efficiency of HRR in cells lacking each protein. Initial studies used etoposide to induce DSBs, followed by analysis of γ-H2AX as a marker of DSB levels. Representative immunofluorescence images are shown in Fig 2A and the % of cells with >5 H2AX foci quantified in Fig 2B. Control cells showed 5, 14 and 45% γ-H2AX-positive cells at 1, 5 and 20µM etoposide, respectively. In cells lacking MCPH1, the % of γ-H2AX-positive cells increased to 19, 35 and 57%, whilst in BRCA2-deficient cells, this increased further to 27, 63 and 83%. Two-way ANOVA revealed that differences were statistically significant (siCON vs siMCPH1 p = 0.0014 and siMCPH1 vs siBRCA2, p = 0.0033).

To specifically investigate HRR efficiency, a DR-GFP assay was used. Briefly, cells contain two non-functional copies of the GFP gene, with one containing a consensus sequence for I-SceI endonuclease. Upon expression of I-SceI (via doxycycline induction), a DSB is induced in one copy of the GFP gene and cells use the other copy of GFP to repair this damage via HRR. Therefore, the % of cells with GFP expression gives a measure of HRR efficiency. Representative flow cytometry profiles are shown in Fig 2C (see section highlighted by red box). In control cells, induction of I-SceI caused ~6.4% GFP-positive cells – this reduced to ~2.9% and ~0.64% in cells lacking MCPH1 or BRCA2, respectively. Combined data from three independent experiments revealed that HRR efficiency was decreased by around 45% in MPH1-deficient cells (p = 0.0028) and >80% in BRCA2-deficient cells (p < 0.001) compared to control cells. The difference in HRR observed between siMCPH1 and siBRCA2 cells was also statistically significant (p = 0.02). Cell cycle analysis confirmed that the % of S + G2 cells (thus with the potential to perform HRR) was not altered by MCPH1 knockdown (data not shown), consistent with published literature [27].

Together, these data suggest that cells lacking MCPH1 are partially defective in the HRR-mediated repair of DNA DSBs but a greater effect is observed in cells lacking BRCA2. In addition, these data suggest that BRCA2 retains some functionality in the absence of MCPH1.

### Differential PARP-1 inhibitor sensitivity in MCPH1- and BRCA2-deficient cells

**Fig 3:**
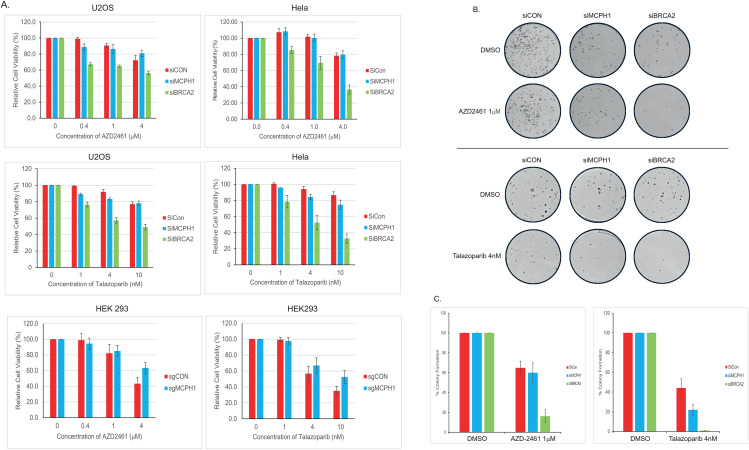
Continued.

Cells lacking HRR can be specifically killed by treatment with PARP-1 inhibitors. Given the differential defect in HRR observed in cells lacking MCPH1 or BRCA2, we investigated how this impacted PARP-1 inhibitor sensitivity. Hela or U2OS cells were depleted of either MCPH1 or BRCA2 using siRNA, before being treated with the PARP-1 inhibitors, AZD-2461 or Talazoparib for 96-hours. Both are potent inhibitors of PARP-1 activity, though Talazoparib is considered a better ‘PARP-1 trapper’. As shown in Fig 3A, MCPH1-deficient U2OS cells treated with AZD2461 showed no significant difference in cell death compared to control cells (p = 0.59), whereas BRCA2 cells were more markedly more sensitive (p < 0.001). Similar results were obtained in Hela cells, with p-values of 0.94 and <0.001, respectively (siCON vs siMCPH1 and siCON vs siBRCA2). After treatment with Talazoparib, MCPH1-deficient cells did show sensitivity compared to control cells (p = 0.02 and 0.002 for U2OS and Hela cells, respectively). Though statistically significant, it should be noted that the differences observed were relatively small. For example, the greatest effect was in Hela cells treated with 10nM Talazoparib, where control cells showed 86% viability compared to 74% in cells lacking MCPH1. To put this into context, the same treatment in cells lacking BRCA2 reduced cell viability to 32%. This is a general trend across all Talazoparib treatments in both cell lines, where BRCA2-deficient cells were markedly more sensitive compared to MCPH1-deficient cells. To confirm that MCPH1-deficient cells showed little or no sensitivity to PARP inhibition, the PARP inhibitor studies were repeated in CRISPR-generated MCPH1 knockout cells (sgMCPH1) and control cells (sgCON). As shown in Fig 3A, sgMCPH1 cells were not more sensitive to AZD2461 or Talazoparib compared to sgCON cells (p = 0.37 and 0.16, respectively).

To further validate these findings, cell viability was investigated using the colony formation assay in Hela cells treated with AZD2461 or Talazoparib. Cells were depleted of MCPH1 or BRCA2, seeded at low density and treated with PARP-1 inhibitor. Colonies were then allowed to form over the course of 10−14 days and stained/counted. Representative images are shown in Fig 3B. Quantification of these colonies revealed that AZD2461 caused no significant sensitivity in MCPH1-deficient cells (p = 0.11) but caused marked sensitivity in BRCA2-deficient cells (p = 0.006, Fig 3C). After Talazoparib, more cell death was observed in cells lacking MCPH1 compared to control (p = 0.11), with an even greater sensitivity observed in BRCA2-deficient cells (p = 0.009). Whilst the siCON to siMCPH1 comparison was not statistically significant, it should be noted that cell viability dropped from ~44% (+/- 9.3) in control cells to ~22% (+/- 5.8) in MCPH1-deficient cells, with larger error bars in the colony assay likely contributing to lack of statistical significance.

Taken together, the MTS and colony data both showed that cells lacking MCPH1 were not sensitive to AZD2461 but did show limited sensitivity to Talazoparib in some scenarios. In contrast, BRCA2-deficient cells showed consistent sensitivity to both PARP-1 inhibitors, with far greater cell death compared to MCPH1-deficient cells.

### Co-Depletion of MCPH1 and BRCA2 does not Increase PARP-1 inhibitor sensitivity

**Fig 4:**
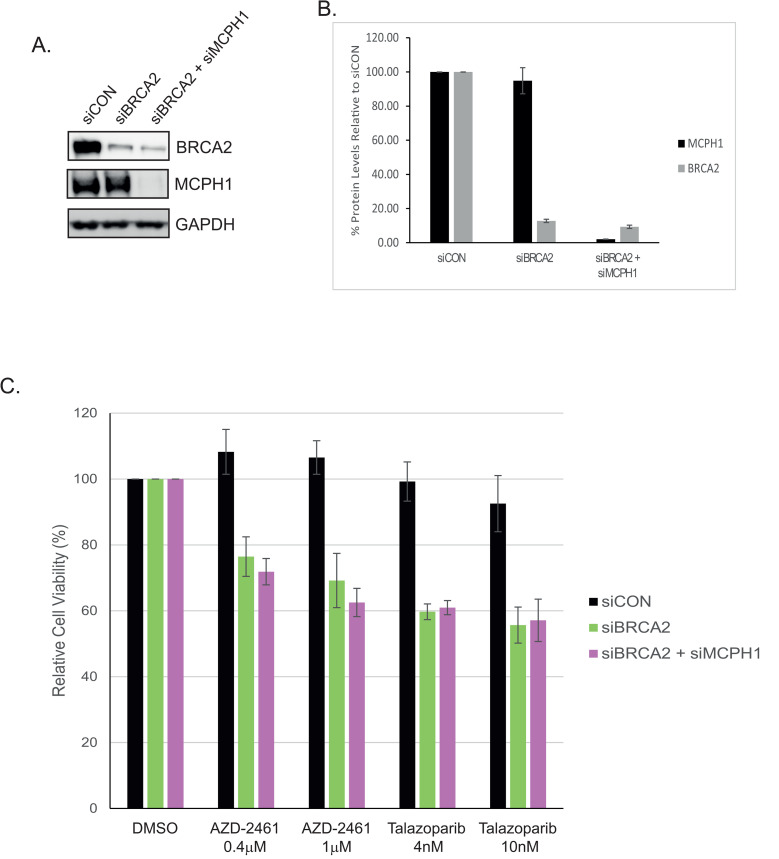
Co-Depletion of MCPH1 and BRCA2 does not enhance PARP-1 Inhibitor Sensitivity. U2OS cells were transfected with control (siCON), BRCA2 (siBRCA2) or BRCA2 + MCPH1 (siBRCA2 + siMCPH1) siRNA for 48h before **(A)** Western blot analysis or (B) seeding into 96-well plates and treating with the indicated doses of AZD2461 or Talazoparib, with each treatment performed in triplicate. After 96-hours, cell viability was measured using an MTS assay. MTS data represents the mean and standard error from at least three independent experiments, with cell viability expressed as a % relative to the corresponding untreated sample. Statistical significance was measured using two-way ANOVA (p-values described in text, with significance set at p < 0.05).

MCPH1- and BRCA2-deficient cells both showed impaired HRR, though the magnitude of defect was far greater in cells lacking BRCA2. We therefore explored whether co-depletion of both proteins caused enhanced PARP-1 inhibitor sensitivity compared to BRCA2-alone. BRCA2 protein levels were depleted by around 90% in both siBRCA2 and siBRCA2 + siMCPH1 cells, whilst MCPH1 was decreased by >95% in the co-transfected cells (Fig 4A and 4B). As demonstrated previously, BRCA2-deficient cells showed a marked and consistent sensitivity to AZD2461 and Talazoparib (p ≤0.002, Fig 4C). This effect was not enhanced in cells that also lacked MCPH1 (p = 0.38 and 0.78, respectively).

## Discussion

This study aimed to investigate whether MCPH1 deficiency in cancer might be targeted therapeutically and focused on the MCPH1-BRCA2 functional interaction in the context of (i) assessing HRR activity and (ii) PARP-1 inhibitor sensitivity. Our data showed that cells lacking MCPH1 have decreased HRR (~45%) but little/no sensitivity to PARP-1 inhibition, whereas cells lacking BRCA2 have a greater HRR defect (~80%) and a corresponding sensitivity to PARP-1 inhibition, which was not enhanced upon co-depletion of MCPH1. Our study also suggests that BRCA2 retains significant functionality in cells lacking MCPH1. This study focuses on Hela and U2OS cancer cell lines – it would be interesting to investigate the generality of these findings in the context of other tumour types.

MCPH1 is an attractive therapeutic target as (i) it is down-regulated/mutated in several tumour types and (ii) has reported roles in HRR, defects in which can be specifically targeted with PARP-1 inhibitors. For example, analysis of the TCGA database revealed that MCPH1 deletion was common in cancer, with a frequency of 5–15%, depending upon the anatomic site [28]. In terms of specific tumour types, MCPH1 expression was down-regulated in breast (29%), ovarian (33%) and prostate tumours [4,20,21]. Interestingly, a recurrent heterozygous mutation in MCPH1 has also been identified in a Northern Finnish population that causes MCPH1 truncations, genomic instability and predisposes individuals to breast cancer (c.904_916del) [29]. This mutation has been detected in other populations, though at a much lower frequency. Functionally, cells lacking MCPH1 have reported defects in HRR, mediated by lack of BRCA2-RAD51 recruitment/retention to DNA DSBs. Wu *et al* (2009) showed that the MCPH1 C-terminal BRCT domains interacted with BRCA2 and this interaction was needed for the recruitment of BRCA2 and RAD51 to DSBs, though no quantitative data on the magnitude of the defect was reported. The same study also showed that lack of MCPH1 did not affect the BRCA2-RAD51 complex. A similar defect in BRCA2/RAD51 foci was reported in MCPH1 knockout mouse embryonic fibroblasts (MEFs) after IR (in both a p53 wild-type and p53-null background) [12,30]. Finally, MCPH1 was found to directly bind to RAD51 using purified protein pull-down assays [13]. Using a cell-free system, this same study showed that MCPH1 promoted the retention of RAD51 on ssDNA and stabilised RAD51 nucleofilaments. However, it should be noted that this system lacks the regulatory complexities of cell-based chromatin structure and the HRR pathway after DSBs. Our data shows a HRR defect in MCPH1-deficient cells, consistent with previously published studies and consistent with a defect in BRCA2-RAD51. However, the magnitude of HRR defect was far less than that observed in BRCA2-deficient cells. This suggests that BRCA2 retains significant functionality in the absence of MCPH1 and further quantitative experiments are required to clarify this issue, particularly in human cells.

Despite the evidence for MCPH1 being down-regulated in cancer and a participant in HRR, there are limited studies investigating PARP-1 inhibitor sensitivity. For example, Liang *et al* (2020) showed that liver cells lacking MCPH1 had a HRR defect of ~80% and this rendered cells sensitive to Olaparib [31]. Interestingly, in our MCPH1-deficient cancer cell model (U2OS Osteosarcoma), we observed much less of an impact on HRR (with cells retaining >50% activity) and little to no sensitivity to two different PARP-1 inhibitors. It might be that MCPH1 function in HRR varies depending upon cell type. Consistent with this, Wood *et al* (2008) assessed HRR in MCPH1 knockout MEFs and demonstrated a decrease of around 55%. It is also interesting to note that PARP-1 inhibition did cause increased cell death in our studies when the HRR defect was further decreased to >80%, as observed in BRCA2-deficient cells. One interpretation is that there is a threshold for HRR activity, below which cells become sensitive to PARP-1 inhibition. In support, an elegant study investigated PARP-1 inhibitor sensitivity in patient-derived breast cancer xenograft models and correlated sensitivity with commonly-used markers of HRR-deficiency, including HRR gene sequencing, BRCA1 methylation, RAD51 foci and a clinically-utilised kit to assess HRR status [myChoice HRD (homologous recombination-deficient) test, Myriad]. Only RAD51 foci was able to perfectly predict PARP-1 inhibitor sensitivity and, importantly, sensitivity was only observed when the % of cells with >5 foci was below 10% - i.e., xenografts with a partial defect in RAD51 foci were not sensitive to PARP-1 inhibition [32].

Our findings add to the debate regarding how to select patients that will respond positively to PARP-1 inhibitors. PARP-1 inhibitors are currently approved for treating BRCA1/2-mutant ovarian, breast, pancreatic and prostate tumours in specific clinical settings [16–19], though there remains controversy regarding tumours with mutations in other HRR components. For example, the PROfound study demonstrated improved radiologic progression free survival (rPFS) in BRCA1-/BRCA2-mutant metastatic castration-resistant prostate cancer patients after treatment with Olaparib [hazard ratio (HR) 0.34]. In contrast, a sub-group with ATM mutations did not show any improvement in rPFS (HR 1.04). The same trial also performed an exploratory analysis of the impact of other HRR mutants (BARD1, BRIP1, CDK12, CHEK1, CHEK2, FANCL, PALB2, RAD51B, RAD51C, RAD51D or RAD54L), which showed limited Olaparib efficacy (HR 0.88) [16]. Similar results were obtained in HRR-negative metastatic breast cancer, where BRCA1, BRCA2 or PALB2 mutants responded positively to Olaparib, with no response observed in patients with mutations in ATM or CHK2 [24].

As well as clinical studies showing inconsistent response of some HRR-mutant tumours to PARP-1 inhibition, there are similar findings from in vitro studies. For example, conflicting studies in prostate cells lacking ATM have recently been published, with Rafiel *et al* (2020) showing intact HRR and no sensitivity to Olaparib [33]. In contrast, Neeb *et al* (2021) generated a panel of ATM-disrupted clones and did show sensitivity to Rucaparib but only in some clones [34]. Cells lacking BRIP1 (a BRCA1-associated DNA helicase that promotes HRR) did not show sensitivity to three different PARP-1 inhibitors [35]. Finally, an elegant study in 2019 constructed an isogenic panel of DT-40 cells with homozygous deletions in ten different HRR-linked genes. They screened these cells for mutational signatures that could be used to identify HRD-positive cells that respond to PARP-1 inhibition. As part of their work, they investigated cytotoxicity after Olaparib and Talazoparib. Interestingly, they found that BRCA1/2 disruption caused greatest sensitivity, with less sensitivity observed in RAD51-deleted cells and variability in the response of other HRR-deleted genes [36]. Whilst the group did correlate these sensitivities with HRR mutational signatures, it would have been very interesting to directly measure HRR activity (e.g., via RAD51 foci or DR-GFP assay) in this isogenic panel of HRR mutants. Such a systematic analysis would also be very interesting in human cells, though is complicated by the lethality of some gene disruptions. When considering the impact of specific HRR components on PARP-1 inhibitor sensitivity, it is also worth noting that there is a tendency not to publish negative findings [37,38], meaning the studies showing no impact might be under-represented in the literature.

Our study used two different PARP inhibitors. AZD2461 is a derivative of Olaparib, designed to avoid resistance mechanisms mediated by p-glycoprotein efflux. Talazoparib is clinicially approved for the treatment of HER2-negative BRCA-mutant breast cancer and HRD castration-resistant metastatic prostate cancer. Whilst both inhibitors are effective inhibitors of PARP-1 activity, Talazoparib is more cytotoxic as a single agent (in comparison to Olaparib) and a more potent PARP trapper than Olaparib, possibly mediated by allosteric effects enhancing PARP-1-DNA interactions [39]. Whilst we saw no evidence for sensitivity of MCPH1-deficient cells to AZD-2461, it is interesting that some effects were observed for Talazoparib (thought these were relatively small and not always statistically significant). This could be caused by the greater PARP trapping activity of Talazoparib, exploiting (albeit to a small extent) the modest HRR defect in cells lacking MCPH1. BRCA2 also plays a role in stabilising stalled replication forks. It has been proposed that this role could contribute to PARP inhibitor cytotoxicity when replication forks collide with trapped PARP-1, though a recent study suggested that HRR-deficiency is the major factor that causes PARP inhibitor sensitivity in cells lacking BRCA2 [40]. It is also important to acknowledge that PARP inhibitors have the potential to inhibit other ADP-ribose transferases (e.g., PARP2 and PARP3) and various cellular kinases. With regard the latter, when screened for binding to a panel of 392 kinases, Olaparib (from which AZD2461 was derived) bound to none and Talazoparib bound weakly to only two. This is in contrast to rucaparib and niraparib, which bound to 37 and 23 kinases, respectively [41].

The data in this study suggest that MCPH1 and BRCA2 both participate in HRR but BRCA2 has a more fundamental role and is a far greater driver of PARP-1 inhibitor sensitivity than MCPH1-deficiency. Findings have significance in terms of basic biology, where we propose that MCPH1 contributes to HRR but is not essential. Our findings also add to the debate regarding the identification of HRD patients that will respond well to PARP-1 inhibition.

## Supporting information

S1 Fig Original Western blot images. Uncropped images for figures 1 and 4 are shown, with the specific figure indicated. Due to the differing nature in molecular masses (e.g., MCPH1 ~ 93kDa and BRCA2 ~ 384kDa), membranes were often cut to allow simultaneous detection of both proteins on the same membrane. Membranes were then carefully reassembled prior to imaging.
(PDF)

S1 File Data used to generate graphs in Figure 1B and 1D. (XLSX)

S2 File Data used to generate graphs in Figures 2B and 2D. (XLSX)

S3 File Data used to generate the graphs in Figures 3A and 3C. (XLSX)

S4 File Data used to generate the graphs in Figures 4B and 4C. (XLSX)
