# Supplementary material for: Differential sensitivity of MCPH1- and BRCA2-deficient cancer cells to PARP-1 inhibition
Source: PLoS One. 2026 Apr 3;21(4):e0345514. doi: 10.1371/journal.pone.0345514 (PMC13048371; doi:10.1371/journal.pone.0345514)

Figure 1A - uncropped MCPH1 blots 48h

3-8% tris acetate gel

Marker = PageRuler Plus pre-stained standard (Thermo Scientific)

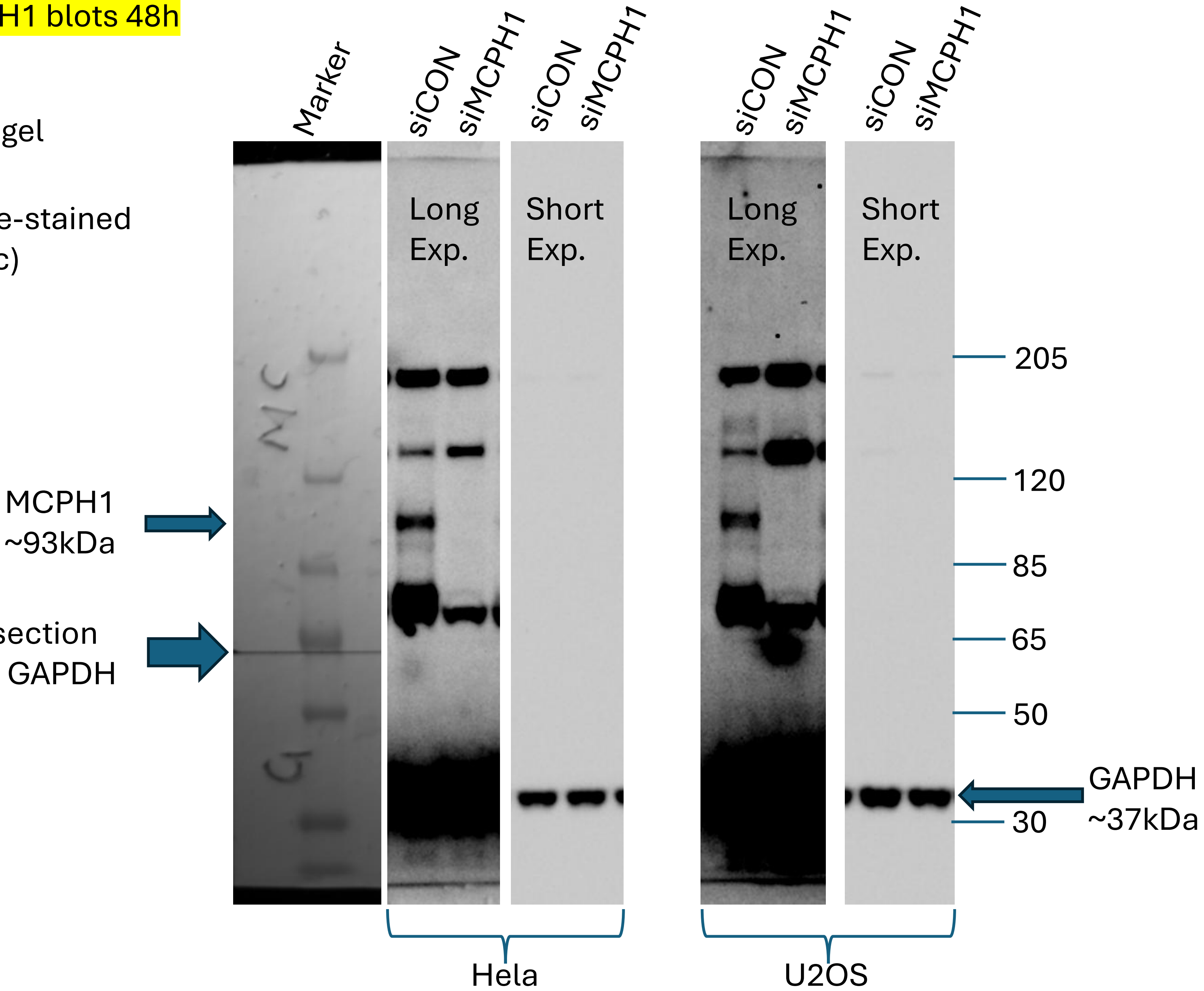

Figure 1A – uncropped BRCA2 blots 48h

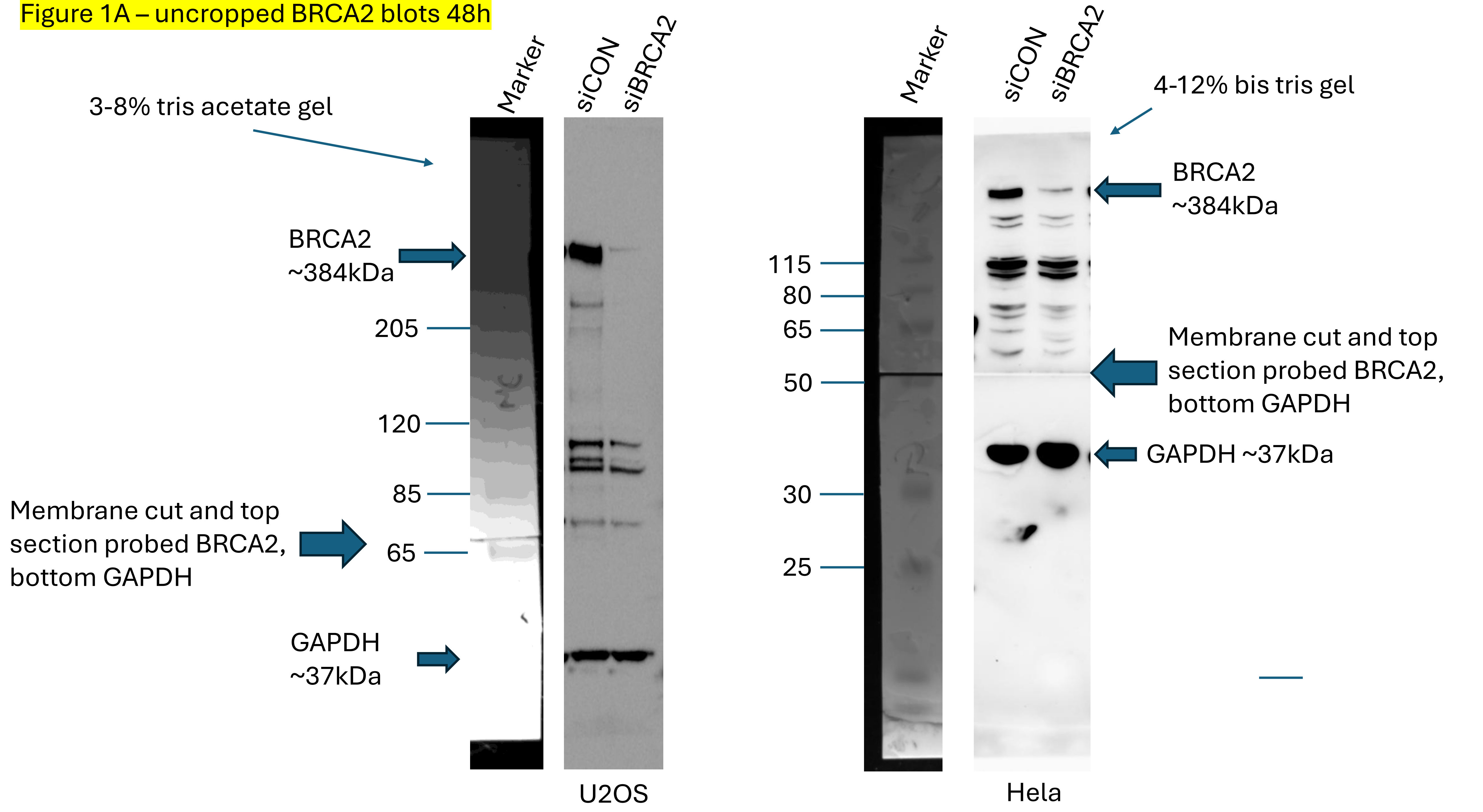

Figure 1A - uncropped MCPH1 blots 120h

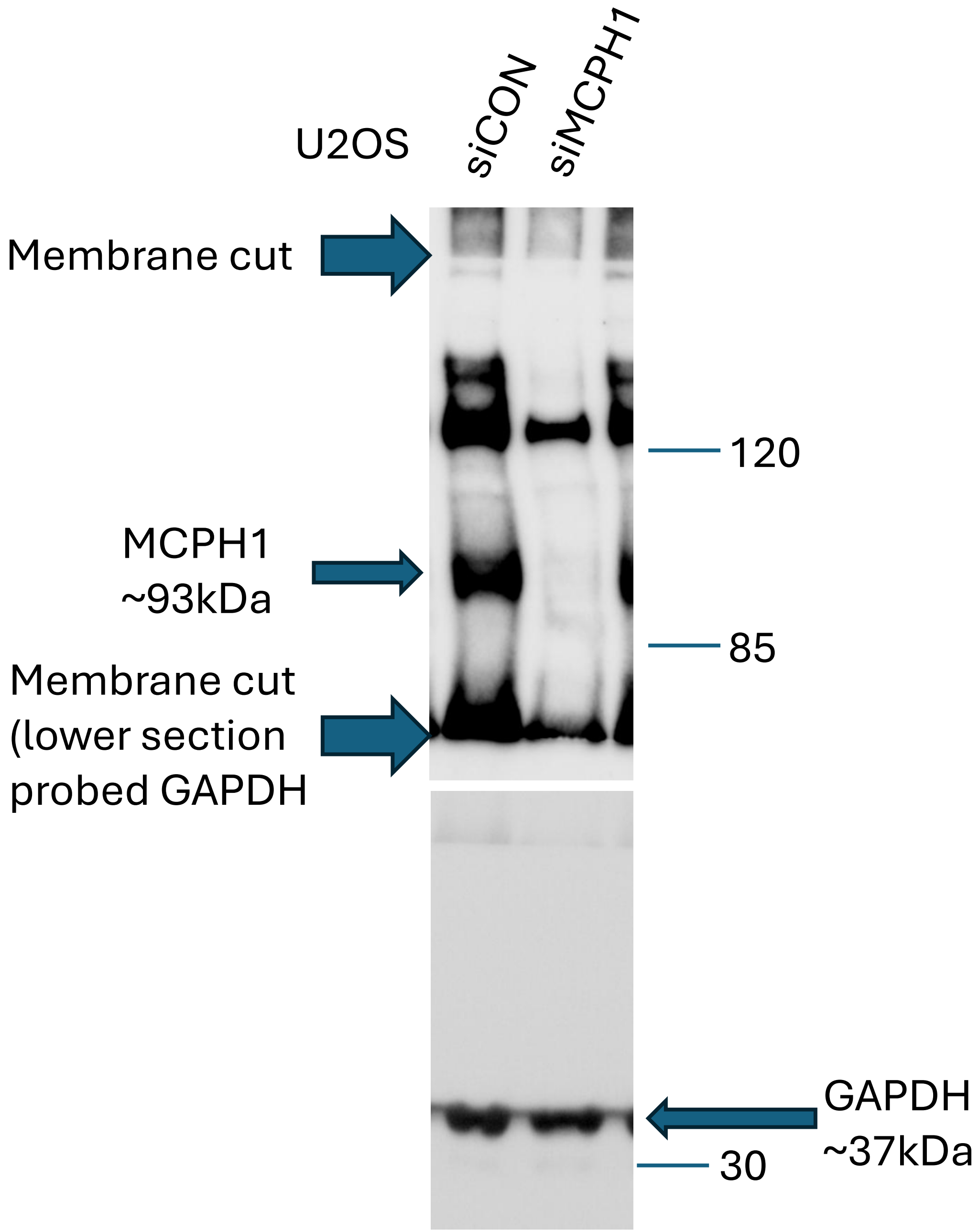

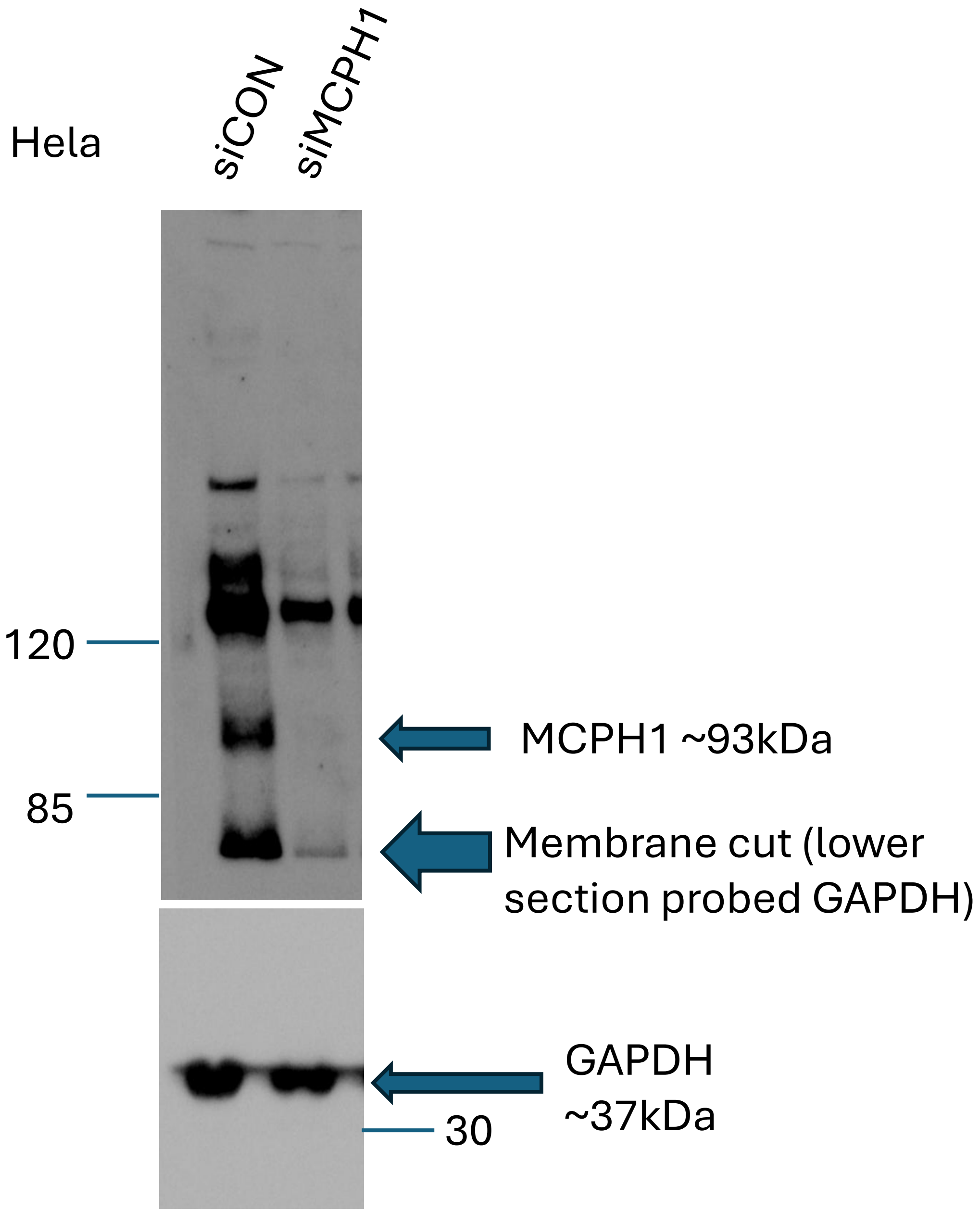

Figure 1A - uncropped BRCA2 blots 120h

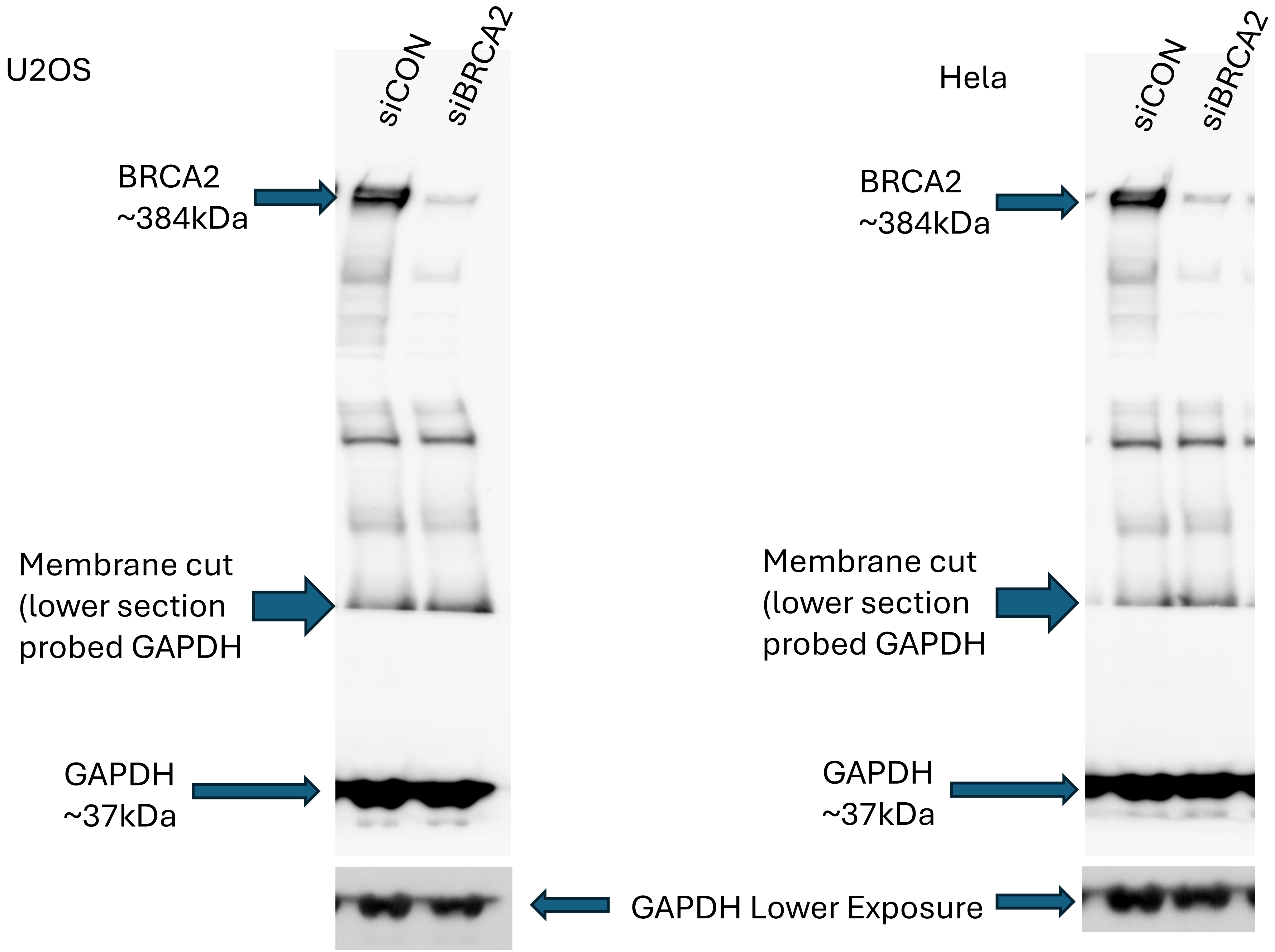

Figure 4A uncropped blots

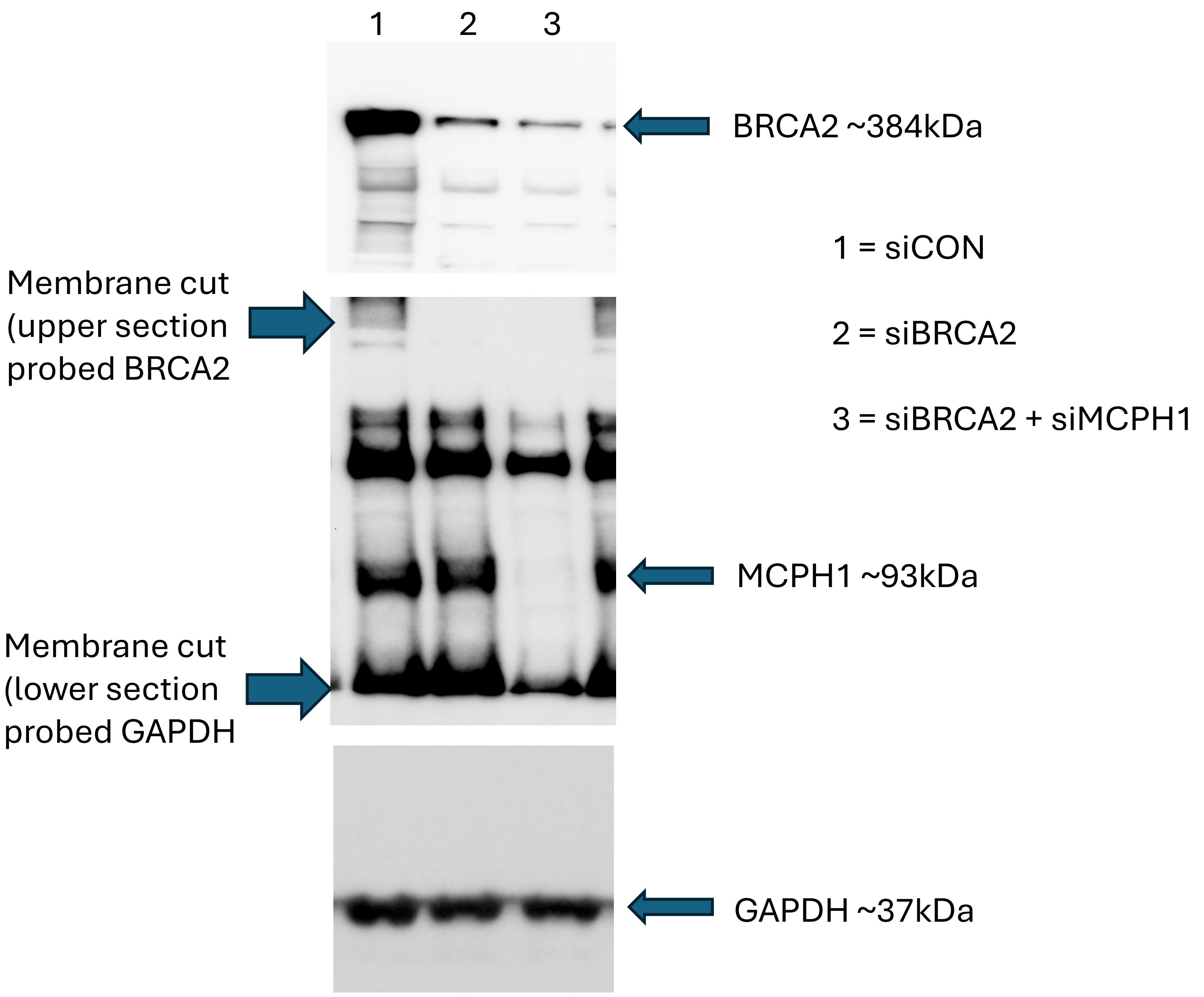

Supplement: S1 Fig — Uncropped images for figures 1 and 4 are shown, with the specific figure indicated. Due to the differing nature in molecular masses (e.g., MCPH1 ~ 93kDa and BRCA2 ~ 384kDa), membranes were often cut to allow simultaneous detection of both proteins on the same membrane. Membranes were then carefully reassembled prior to imaging. (PDF) [file pone.0345514.s001.pdf]
